# Reduced Power in Fronto-Parietal Theta EEG Linked to Impaired Attention-Sampling in Adult ADHD

**DOI:** 10.1523/ENEURO.0028-21.2021

**Published:** 2022-01-05

**Authors:** Benjamin Ultan Cowley, Kristiina Juurmaa, Jussi Palomäki

**Affiliations:** 1 Faculty of Educational Sciences, University of Helsinki, Helsinki 00014, Finland; 2 Cognitive Science, Department of Digital Humanities, Faculty of Arts, University of Helsinki, Helsinki 00014, Finland

**Keywords:** ADHD, adult, attention, EEG, inhibition, rhythmic attention, sustained attention, TOVA

## Abstract

Attention-deficit/hyperactivity disorder (ADHD) in adults is understudied, especially regarding neural mechanisms such as oscillatory control of attention sampling. We report an electroencephalography (EEG) study of such cortical mechanisms, in ADHD-diagnosed adults during administration of Test of Variables of Attention (TOVA), a gold-standard continuous performance test for ADHD that measures the ability to sustain attention and inhibit impulsivity. We recorded 53 adults (28 female, 25 male, aged 18–60), and 18 matched healthy controls, using 128-channel EEG. We analyzed sensor-space features established as neural correlates of attention: timing-sensitivity and phase-synchrony of response activations, and event-related (de)synchronization (ERS/D) of α and θ frequency band activity; in frontal and parietal scalp regions. TOVA test performance significantly distinguished ADHD adults from neurotypical controls, in commission errors, response time variability (RTV) and d′ (response sensitivity). The ADHD group showed significantly weaker target-locked and response-locked amplitudes, that were strongly right-lateralized at the N2 wave, and weaker phase synchrony (longer reset poststimulus). They also manifested significantly less parietal prestimulus 8-Hz θ ERS, less frontal and parietal poststimulus 4-Hz θ ERS, and more frontal and parietal prestimulus α ERS during correct trials. These differences may reflect excessive modulation of endogenous activity by strong entrainment to stimulus (α), combined with deficient modulation by neural entrainment to task (θ), which in TOVA involves monitoring stimulus spatial location (not predicted occurrence onset which is regular and task-irrelevant). Building on the hypotheses of θ coding for relational structure and rhythmic attention sampling, our results suggest that ADHD adults have impaired attention sampling in relational categorization tasks.

## Significance Statement

This study identifies one factor potentially contributing to difficulty of paying sustained attention among adults with attention-deficit/hyperactivity disorder (ADHD). We recorded electroencephalography (EEG) from a good-sized sample of adults with ADHD diagnosis (*N* = 53), while they performed a gold-standard computerized performance test (CPT), Test of Variables of Attention (TOVA). We found that, compared with matched healthy controls (*N* = 18), ADHD participants showed reduced EEG indices of strength of rhythmic attention-sampling. The primary such index was parietal θ-rhythmic event-related synchronization (ERS), while other indices related to amplitude and phase synchrony of neural responses to stimuli. Characteristics of the task and brain-signal analysis suggest that ADHD participants may be deficient in relational processing, which (to our knowledge) has not been shown before.

## Introduction

Attention-deficit/hyperactivity disorder (ADHD) is a common childhood psychiatric disorder, marked by persistent, age-inappropriate levels of inattention and hyperactivity/impulsivity, which persists into adulthood in approximately one third of those diagnosed (Swanson et al., 1998). Here, we investigate how the neural correlates of attention in adults diagnosed with ADHD differ from healthy controls.

ADHD is characterized by deficits in completing tasks that challenge long-lasting self-regulation of attention. One influential model says that ADHD is associated with deficient inhibitory control, which affects other executive functions such as working memory (Barkley, 1997). Thus, study of ADHD must elicit interplay between inhibition, vigilance/inattention, and sustained attention, and examine the underpinning neural processes, such as regulation of perceptual processing by (dorsal or ventral) attention networks (Spyropoulos et al., 2018).

### Attention testing

**Figure 1.**
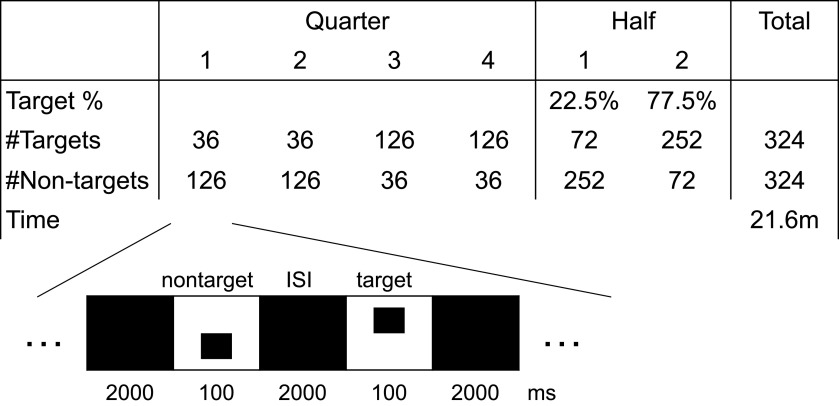
TOVA experiment protocol design. Above, Number of targets and nontargets in each quarter and half, with target-to-nontarget frequency and total time. Below, Individual trial structure, millisecond timings, and stimuli appearance.

One protocol that probes inhibition, inattention, and sustained attention is Test of Variables of Attention (TOVA), which has good external validity as it is widely used as a ‘gold standard’ to detect attention problems typical for ADHD. TOVA is a computerized performance test (CPT) with a monotonous hybrid Go/NoGo target-classification task design (Leark et al., 2007; Strauss et al., 2006): participants must respond to targets and inhibit response to nontargets (Fig. 1). Many behavioral studies have used TOVA to test ADHD-diagnosed individuals, primarily children (González-Castro et al., 2016; Rodríguez et al., 2016). Far fewer studies have measured neural activity during TOVA (Halawa et al., 2017; Keith et al., 2015), and none studied adults with ADHD. Although TOVA was not designed as a cognitive neuroscience protocol, its status in clinical use makes it a compelling task for study of neural mechanisms of ADHD.

### Neural mechanisms

In the scientific literature, there is an ongoing search for aberrant neurocognitive mechanisms leading to ADHD symptomatology. Observed deficits in attention, inhibition, and working memory suggest that activity in the frontal cortico-striatal network and frontoparietal attention network (FPN) are altered in ADHD (Loo et al., 2007), and may be linked to aberrant default mode network (DMN) suppression (Sonuga-Barke and Castellanos, 2007), or α band oscillation suppression (Lenartowicz et al., 2018). Both volumetric alterations and changes in task-dependent fMRI signals in prefrontal-striatal circuits support this view (Castellanos and Proal, 2012). Mowinckel et al. (2017) also found causal support for these ideas from fMRI functional connectivity analysis of a decision-making task in a methylphenidate intervention.

Cortical oscillations play a fundamental role in brain networks (Klimesch, 1999) and have been shown to be altered in ADHD participants (Calderone et al., 2014), especially in FPN. Decline in sustained performance has been associated with changes in the ratio of frontomedial θ (4–8 Hz) to occipital α (8–12 Hz) power (Clayton et al., 2015). This could be because of reduced attentional sampling (at θ frequencies) and DMN suppression (at α frequencies), driven by neuroenergetic fatigue (Killeen, 2013).

In general, α oscillations in task-relevant areas tend to reduce in amplitude (because of neural desynchronization) during effortful cognitive processing (Clayton et al., 2015). The functional role of α has been proposed as attention gating by oscillatory inhibition (Jensen and Mazaheri, 2010), and as control of memory access (Klimesch, 2012). α synchronization of frontal, parietal, and visual regions has been shown to facilitate visuospatial attention (Lobier et al., 2018). In adults with ADHD, weaker posterior α modulations have been observed (Hasler et al., 2016), and α synchronization is attenuated according to a recent review (Lenartowicz et al., 2018).

In contrast to α, θ amplitudes tend to increase with increasing cognitive effort and mental fatigue (Sauseng and Klimesch, 2008; Klimesch, 2012; Wascher et al., 2014), which may be linked to cognitive demand from monitoring of task-relevant activities and from cognitive control, both implicated in sustained attention (Cavanagh and Frank, 2014; Clayton et al., 2015). The role of θ (integrated with γ with which it co-occurs) has been proposed as arranging item representations in a relational code (Lisman and Jensen, 2013).

### Oscillatory versus evoked activation

Oscillations can become entrained to predictable stimulus onsets, i.e., oscillatory phase becomes more likely to coincide with the expected stimuli (Arnal and Giraud, 2012). This entrainment can be observed already in the prestimulus period (Lakatos et al., 2008; Calderone et al., 2014), indeed several studies have linked entrainment of prestimulus α oscillations to fluctuations in visual awareness and sustained attention (Thut et al., 2006; Hanslmayr et al., 2007; Mathewson et al., 2009; Busch and VanRullen, 2010; Macdonald et al., 2011; VanRullen et al., 2011; Hamm et al., 2012). Given the weaker α seen in ADHD adults, this may explain why they tend to be ill-prepared (under lab conditions) for incoming stimuli that may require a response (Russell et al., 2006).

Study of oscillatory neural mechanisms requires high time-resolution, magneto- or electroencephalography (M/EEG) recording. However, a sudden-onset trial-based task like TOVA presents the challenge of analyzing oscillations in the presence of strong evoked signal power, i.e., power locked to stimulus onset phase. However, because TOVA presents stimuli periodically, with little variation in visual characteristics of each condition, we expect that a visible predictive processing (Sherman et al., 2016) based endogenous signal will be evident. In other words, we suggest that observed differences in group brain activity reflect endogenous oscillatory activity interacting with exogenous activity from evoked responses; therefore, TOVA can also be used to study oscillations.

### Research questions (RQs)

Here, we aim to study behavioral performance and EEG-recorded evoked potentials and cortical oscillations during TOVA, comparing adults with ADHD to healthy controls. In particular, we examine how frontal and parietal neural correlates of visual attention differ between groups throughout TOVA. We focus on the following RQs:

RQ1: How do ADHD and control groups differ in EEG amplitude response to targets during TOVA?

RQ2: How do groups differ in phase synchrony of EEG amplitude to TOVA trials?

RQ3: How do groups differ in the temporal dynamics of frequencies linked to attentional regulation, α and θ?

RQ4: How do neural correlates of attention evolve over the duration of TOVA?

## Materials and Methods

To address our RQs, we examine frontal and parietal regions of interest (ROIs), using time-frequency representations (TFRs), event-related potentials (ERPs), and phase-synchrony measures. We test interactions of group with TOVA trial conditions (response to target vs inhibit response to nontarget) as they vary across TOVA test conditions [first half (H1) infrequent vs second half (H2) frequent target mode]. Data measurement consisted of visual TOVA CPT with EEG recording, gathered as part of a larger project detailed in (Cowley et al., 2016). An ethical approval was obtained from the ethical committee of the hospital district of Helsinki and Uusimaa. Each participant gave informed consent in accordance with the Declaration of Helsinki.

### Participants

We recruited 53 adults (25 males, age mean (M) = 36.26, SD = 10.22) diagnosed with either ADHD (*n* = 44) or ADD (*n* = 9), we refer to both as ADHD group, and a healthy control group of 18 adults (six males, age M = 32.78, SD = 10.82) with no diagnosed neuro-cognitive deficits or ongoing medication for ADHD/ADD. The healthy controls were significantly more likely to have TOVA performance within normal limits compared with ADHD adults (as detailed in Results). All participants had normal or corrected-to-normal vision. The groups did not differ in terms of age, gender, or handedness. Because of reasons of data quality (detailed below) a number of participants were dropped from EEG analysis for different TOVA conditions. The final analyzed number of participants were: ADHD inhibition H1 41, H2 31; response H1 39, H2 37; control inhibition H1 15, H2 11; response H1 14, H2 13. The control group size was always at least 35% of the size of the ADHD group, and thus the statistical power of our tests is not substantially affected by the between-group size disparity (see https://www.markhw.com/blog/control-size for a simulation analysis which suggests that it is reasonable to use a control group 30% as large as the test group).

For the current study, inclusion criteria for the ADHD group were (1) preexisting diagnosis of ADHD/ADD, (2) no neurologic diagnoses, (3) age between 18 and 60, (4) scores on Adult ADHD Self Report Scale (ASRS; Kessler et al., 2005) and Brown ADHD Scale (BADDS; Brown, 1996), indicating the presence of ADHD, and (5) an IQ score of at least 80 using WAIS IV measured by a qualified psychologist (Wechsler, 2008). No strict cutoff values were used for ASRS and BADDS to indicate the presence of ADHD/ADD. Instead, exclusion was decided by the consulting psychiatrist, who conducted structured clinical interviews with participants as per the guidelines of the Diagnostic Interview for ADHD in Adults (DIVA 2.0; Kooij and Francken, 2010). Comorbidities were evaluated during the clinical interview, and exclusion criteria included outlier scores in scores of Generalized Anxiety Disorder (Spitzer et al., 2006), Beck Depression Inventory (Beck et al., 1996), Alcohol Use Disorders Identification Test (Saunders et al., 1993), the Mood Disorder Questionnaire (Hirschfeld et al., 2000), test of prodromal symptoms of psychosis (Heinimaa et al., 2003), and the Dissociative Experiences Scale (Liebowitz, 1992). The psychiatrist made a final assessment of the balance of symptoms contributing to patients’ presentation.

Within the ADHD group, there was a small difference in the ASRS hyperactivity-impulsivity scores depending on the diagnosis (*F*_(1,50)_ = 5.01, *p* < 0.030, *r*^2^ = 0.09). As expected, participants with hyperactive-impulsive (ADHD-HI) or combined (ADHD-C) diagnoses had higher hyperactivity-impulsivity scores (M = 6.33, SD = 2.56; the groups were combined) than those with an inattention (ADHD-I) diagnosis (i.e., without hyperactivity-impulsivity; M = 4.22, SD = 2.59). There was no significant difference in the ASRS inattention scores between groups, and most analyses treat all ADHD participants as one group, except where noted below.

### Procedure

The behavioral and EEG data were measured in a 2.5- to 3-h multitask session, gathered in an electrically-shielded and sound-attenuated room. TOVA was administered toward the beginning of the measurement session (as suggested by Greenberg et al., 2016; p. 24). The full session included preparation (30–40 min), pretest baseline measurement (5 min), TOVA (22 min), resting state vigilance measurement (20 min), a novel CPT (30 min; detailed in Cowley, 2018), and a post-test baseline measurement (2 min).

Participants were asked to abstain from taking their ADHD medication for 48 h before the EEG measurement (washout period). They were also advised not to take any other stimulants immediately before the measurement (e.g., coffee, cigarettes, energy drinks) and to arrive as well rested as possible to the measurement.

Karolinska sleepiness scale (KSS; Akerstedt and Gillberg, 1990) was employed to control for participants’ sleepiness levels, as sleepiness can affect both sustained attention and EEG measurement. Participants tended to report being alert (on the scale 1–9, ADHD: M = 4.60, SD = 1.38, controls: M = 3.73, SD = 1.39; these mean scores indicated 3 “alert,” to 4 “rather alert,” and 5 “neither alert nor sleepy”).

### TOVA

We administered TOVA visual version 8, consisting of the test software, USB relay hardware, a low-latency microswitch hardware response button, and Synchronization Interface hardware for test-to-EEG amplifier synchrony (all products of the TOVA Company).

TOVA presents a target and a nontarget stimulus, shown in Figure 1. The participant’s task is to respond to targets by a button press of their dominant hand, and refrain from responding to nontargets, i.e., it has a Go/NoGo design. The participants are advised to respond as accurately and quickly as possible. A practice test of ∼20 trials is administered before the actual test. Trials can be: correct responses, commission errors (incorrect responses), omission errors (incorrect inhibition), and correct inhibition.

TOVA has two consecutive conditions: infrequent and frequent target mode. During H1, the target stimulus appears infrequently (72 targets, and 252 nontargets, i.e., 22.5% target probability). During H2, these frequencies are reversed (252 targets and 72 nontargets, i.e., 77.5% target probability). Participants are not informed about this transition. Each stimulus is presented for 100 ms and separated from the next stimulus by a 2000-ms interstimulus interval (ISI). The anticipatory cutoff time from stimulus onset is 150 ms, meaning that responses given 0–150 ms after stimulus onset are considered invalid. The duration of TOVA is 21.6 min, each condition lasting 10.8 min.

### TOVA variables

Analysis was performed on TOVA’s “standard scores,” which are formed by comparing individual performance against a normed sample population (*N* = 1596), this implies that behavioral variables do not represent, e.g., actual error but rather the difference from a “normal” value for error (Greenberg et al., 2016). The comparison group for each participant is determined by their age group and gender. TOVA software performs normative classification of test subjects’ performance as “not within normal limits,” “borderline,” or “normal.”

Five standard scores are available: (1) mean response time (RT): mean of correct RTs in milliseconds (ms); (2) RT variability (RTV): SD of mean correct RT; (3) commission errors: incorrect responses to a nontarget stimulus; (4) omission errors: incorrect responses to a target stimulus; and (5) d*^′^*: ratio of hits (correct responses) to false alarms (commission errors). One participant who showed strong evidence of symptom exaggeration (Symptom Exaggeration Index; SEI) was excluded from further analyses.

For normative comparison, TOVA is advised to be measured between 6 A.M. and 1 P.M. Our group-average measurement times were close to or within the normed hours: ADHD M = 1:45 P.M., SD = 2 h 26 min (35 after 1 P.M.); control M = 12:49 P.M., SD = 3 h 2 min (7 after 1 P.M.), and the starting times did not statistically differ between the groups.

### EEG measurement and analysis

EEG was measured using Biosemi ActiveTwo equipment with 128 active electrodes mounted on a cloth headcap with equiradial positions. Electrode positions are labeled in text according to the closest International 10–5 electrode placement system label (Jurcak et al., 2007). Active electrode CMS (Common Mode Sense) and passive electrode DRL (Driven Right Leg) were used to create a feedback loop for amplifier reference. Electrooculography (EOG) was recorded using bipolar montage: horizontal EOG electrodes were attached to the outer canthi of both eyes; vertical EOG electrodes were attached above and below the left eye. Electrode offsets (running average of voltage at each electrode) were kept below ±25 mV.

During the TOVA, participants were advised to relax and sit as still as possible, avoiding excessive movements, and to fixate the middle of the screen shown as a small white dot between stimulus presentations. Three participants were excluded because of technical problems with event codes in the EEG data (for the final number of available participants and trials available for each statistical EEG analysis, see table A1 at https://doi.org/10.6084/m9.figshare.13614446.v1).

### Preprocessing

The data were preprocessed using Computational Testing Automated Preprocessing toolbox (CTAP; Cowley et al., 2017; Cowley and Korpela, 2018), based on EEGLAB (Delorme and Makeig, 2004) for MATLAB. The data were re-referenced offline to the average of the two mastoids. The data were then low-pass filtered at 45 Hz and high-pass filtered at 2 Hz. CTAP was used to detect eye blinks using the probabilistic time-domain method detailed in Cowley et al. (2017), the validity of which was examined for each participant by visual inspection.

Each participant’s continuous EEG and EOG data were decomposed using the FastICA algorithm (Hyvarinen, 1999). Independent components (ICs) statistically similar to the CTAP-detected blinks were removed (Cowley et al., 2017). Further, artifactual channels, epochs, and ICs were identified using methods from Fully Automated Statistical Thresholding for EEG artifact Rejection (FASTER; Nolan et al., 2010). Data for automatically rejected channels was interpolated from adjacent electrodes. Fluctuations exceeding ±80 μV in amplitude across >50% were discarded as bad segments. Any remaining ocular-artifact or muscular-artifact ICs were detected and rejected manually by joint visual inspection of IC activations, scalp maps, power spectrum, and ERP image. On average 3% of channels and one IC per participant were rejected.

### Data shaping

For event-related analyses, the EEG data were extracted into four types of epochs: commission errors, correct responses, omission errors and correct inhibition. Epochs lasted 2000 ms, with 1000 ms before and after stimulus onset. Epochs that contained a blink (as detected by CTAP) starting from 400 ms before stimulus onset to 600 ms after stimulus onset, were rejected. After this, too few error trials remained to conduct statistical analyses: on average, controls had seven and ADHD nine trials for commission errors; controls had two and ADHD six trials for omissions. Thus, we analyzed only the correct (inhibition and response) trials.

To counterbalance fluctuations in voltage offset across epochs and participants, baseline correction was applied to each epoch by subtracting the baseline mean amplitude from the whole epoch. For those data where the analysis concerned prestimulus phase, baseline period was from 1000 to 900 ms before stimulus onset. For all other analyses, baseline period was −1000–0 ms.

Epochs were divided between correct responses and inhibits, in H1 and H2, to give four datasets per group for most analyses (see Results below for details of exceptions). For each of these four conditions, a participant was only included if they had at least 36 epochs for infrequent conditions (responses H1 and inhibits H2) or 72 epochs for frequent conditions (inhibits H1 and responses H2). This resulted in the participant numbers given above, and ensured each participant contributed 108 clean epochs per each test half (i.e., H1 had 36 response epochs and 72 inhibit epochs, totaling 108 epochs, and vice-versa for H2). The epochs were randomly sampled without replacement from the total available per condition, on a participant-wise basis. In order to test that this sampling did not bias results, we conducted 11 similar draws and estimated variance across draws of the spectral power (calculated as described three sections below, RQ3: spectral power and TFR calculation), the final mean variance was only 0.015.

The number per participant was fixed to provide balanced data and help ensure that any observed effect would be because of altered neural processing, not merely because of possible differences in behavioral performance (e.g., amount of button presses). In order to validate that any group differences in button presses would not bias neural results, we also tested the behavioral data, as reported in Results, and found no evidence of bias.

Analyses focused on two ROIs (denoted using 10–5 system labels mapped from the Biosemi 128 channel): frontal at F4, F4h, Fz, F3h, F3, AFF4h, AFz, and AFF3h and parietal at P3, P1, CPPz, P2, P4, PO3, POz, and PO4. These locations are illustrated below. The electrode locations were chosen a priori based on previous research (Hanslmayr et al., 2005, 2007; Mathewson et al., 2009; Macdonald et al., 2011), but modified slightly to give balanced and similar coverage frontally and parietally.

### RQ1: ERP calculation

ERP images, displaying trials individually by visualizing amplitude with color, were used to show response-sorted activation within frontal and parietal ROIs (see section RQ1: response amplitude). These images were generated by ordering trials according to their RT value, and smoothing across trials with a moving Gaussian of width proportionate to the number of trials (Gaussian SD = trial number/30 ⇒ window width = trial number/5). Data were twice re-epoched to 1-s windows containing target and response, in one case target-locked and the other response-locked. Corresponding ERP waves were computed twice: adjacent to the ERP image are plotted the grand average ERPs of trials with RT less than, and greater than, median RT. Below the ERP images are plotted comparisons of grand-average ERPs for control and ADHD, with amplitude testing shown (see Statistical Analysis section).

### RQ2: phase-related calculations

We also plotted phase-sorted ERP images and intertrial coherence (ITC; see Fig. 5). Both these analyses focused on 10 Hz, not only because it is the canonical value of peak α and prominent parietally in both group’s spectrograms (see Fig. 6), but also the timing of TOVA stimuli is 10 Hz, i.e., stimuli are shown for 100 ms per trial.

Phase-sorted ERP images were computed for target and nontarget trials, in both ROIs, and show only the first 400 ms poststimulus (where coherent waves are prominent). Data were further subset (by random sampling without replacement) to ensure that each condition had a proportionate number of trials (see Fig. 5*A*,*B*). Phase sorting was conducted in EEGLAB, computing the phase at −80 ms for the frequency with maximum power in the α band 8–12 Hz, and retaining the 95% of trials with largest power for plotting. Sorted trials were smoothed with a moving Gaussian of width proportionate to the number of trials (Gaussian SD = trial number/30, window width = trial number/5)

We used EEGLAB to compute 10 Hz α parietal ITC, separately for the two TOVA halves (H1 and H2) and two groups, shown in Figure 5*C*.

### RQ3: spectral power and TFR calculation

In order to assess how the EEG spectrum changed from prestimulus to poststimulus, oscillatory power was calculated separately within prestimulus and poststimulus periods, using Welch’s power spectral density estimation of the mean log power spectrum via EEGLAB’s spectopo function (see Fig. 6). The FFT window length was 512 (i.e., sample rate), with overlap 384 frames. Power was calculated for each 1-Hz frequency bin from 4 to 16 Hz).

To examine the effect of TOVA condition on prestimulus activity, α power was also calculated for the 500-ms prestimulus period preceding correct trials for TOVA halves H1 and H2, in the parietal ROI. All correct trials (both correct inhibition and correct response) were combined for this analysis, since the focus was prestimulus. Within these correct trials, prestimulus power was calculated for each 1-Hz frequency bin separately within the extended α band (8–12 Hz).

To examine event-related oscillatory power dynamics for correct inhibition and response trials, we calculated three types of TFR: event-related spectral perturbations (ERSP; Makeig et al., 2004), evoked power, and induced power; each within the frontal and parietal ROIs defined above. All three are plotted below showing event-related synchronization (ERS) or event-related desynchronization (ERD) as warm or cold colors, respectively.

A TFR is obtained by calculating spectral power across an epoch, for multiple frequencies. ERSP is defined as the average of TFRs computed for each separate trial, and is sometimes referred to as the total power. Evoked power is the TFR of the time-domain average of all epochs (i.e., the classic ERP). Induced power is defined as the total power minus the evoked power (intended to capture nonphase locked activation), but various methods to calculate it have been discussed (see David et al., 2006; Roach and Mathalon 2008): here, we calculate it by subtracting the grand average ERP from each epoch in time domain, then compute the TFR for each epoch and take their average.

To obtain these power estimates for ROIs, we created custom MATLAB code (based on EEGLAB’s newtimef function) to calculate TFR matrices for each electrode individually and take the mean of all electrodes to obtain the spectral power estimate reported below. This preserves estimates of spectral power from separate electrodes, as opposed to averaging channels first and computing TFR for the average, which may underestimate spectral power for electrodes at different phases.

Spectral power was calculated in 54 frequencies from 4 to 30 Hz, in 200 time points (−583–581 ms), using Morlet wavelets with scaling factor 0.5 (such that cycles scaled linearly from 3 to 11.25, and window size from 427 to 214 samples). Adjusting the wavelet scaling factor along with frequency aimed to balance the trade-off between temporal and spectral resolution. Note, the design of TOVA trials constrained the ERSP calculation to start at 4 Hz, since lower frequencies returned very narrow windows.

Our results (especially Fig. 7) show that ERSPs contain both induced and evoked power, but mainly the former, and illustrate well the systematic time-frequency effects since they reflect a “smoothed” version of spectral power (compared with evoked power). We thus use ERSPs as a preferable way to investigate event-related time-frequency phenomena, and they are also computed in a standard way and comparable between studies.

### RQ4: effect of time

The effect of time in TOVA is confounded by the switch from infrequent to frequent target presentation across TOVA halves, which was not counterbalanced in our data because (at the time of testing) TOVA software was not configurable. Although we obtain some insight from comparing H1 and H2 results, these should be taken with due caution. We thus analyzed the effect of time within-halves, by splitting the datasets for each condition (H1 vs H2 × response vs inhibit) into “early” and “late” trial subsets of equal size (these are not exactly equivalent to Q1 and Q2 because they derive from already randomly sampled subsets, which may skew toward the beginning or end of each half).

### Statistical analyses

#### Behavioral performance

Behavioral performance was analyzed using a 2 × 2 two-way mixed design MANOVA, with group (ADHD vs control) as a between-subjects factor and TOVA condition (infrequent vs frequent) as a within-subjects factor. The DVs were mean RT, RTV, commission errors, omission errors, and d*^′^* (for more details, see https://doi.org/10.6084/m9.figshare.13614446.v1). These were followed up by further DV-specific analyses, which are detailed in the Results. We also assessed how well behavioral performance in TOVA differentiates between ADHD diagnosed adults and healthy controls.

Effect sizes for repeated measures were computed as partial η squared. Multiple comparisons were adjusted with Bonferroni correction, keeping the α at.05. Possible extreme outlier values were assessed by examination of studentized residuals for values greater than ±3. We do note, however, that excluding outlier scores in a clinical sample is not straightforward, as high behavioral variability itself has been reported as a clinical characteristic in adults with ADHD (Castellanos et al., 2005). In total four outlier scores were excluded from the analysis of standardized scores. Excluding them did not affect the interpretation of results, while including them would have violated the assumption of homogeneity of variance-covariance matrices. When necessary, variables with skewed distributions were log-transformed. However, group means and standard deviations are reported in original untransformed format, as these are more meaningful to interpret. The assumptions of repeated measures ANOVA were satisfied on transformed data without outliers.

In order to validate that the neural data analyses would not be confounded by relative variation in motor processing activations, because of any observed between-group difference in amount of button presses (e.g., because of higher commission error rates), we conducted the following analysis. We tested whether there was a difference in total number of button presses (= correct target presses + commission errors) between the treatment groups or test quarters by fitting a robust linear mixed model (LMM) with total presses as the DV, and treatment group (ADHD, control), test quarter (Q1–Q4), and their interaction, as the predictors. A numerical participant ID was used as a random factor, allowing variability in the intercepts but not slopes. Robust LMM (Koller, 2016) was used since the DV was bimodally distributed across test quarter [test quarters are confounded by the TOVA conditions of infrequent (Q1–Q2) vs frequent (Q3–Q4) targets], with far fewer total button presses occurring during Q1 and Q2 than during Q3 and Q4.

#### EEG data

Statistical analyses of EEG were run in MATLAB (version 9.7), using EEGLAB toolbox (Delorme and Makeig, 2004) and in R platform for statistical computing (R Core Team, 2020).

#### RQ1

Group differences in correct-response trial ERP waves were tested using two-sample Kolmogorov–Smirnov test of the mean amplitude within two time windows per trial: 150–250 and 330–430 ms in target-locked, and −170 to −70 and 0 to 100 ms in response-locked trials (see Fig. 3, third row of each panel). These windows were centered on the N2 and P3 waves observed in the data. False discovery rate (FDR) correction (using the procedure described by Storey, 2002) was used to correct for multiple comparisons at the *α* = 0.05 level.

#### RQ2

We computed statistical significance of the ITC using EEGLAB’s permutation-based testing of single-trial spectral estimates across latencies (Delorme and Makeig, 2004), with default parameter settings.

#### RQ3

We analyzed the differences between baseline and poststimulus periods in their log-transformed power spectral density (log-PSD) at 4- and 8-Hz frequency bins (these frequencies were chosen based on their role in attention, and confirmed by visual inspection; see Fig. 6). We fit a Bayesian LMM (Chung et al., 2013) with log-PSD as the DV, condition (baseline, poststimulus), frequency (4, 8 Hz), and group (ADHD, control), and full factorial interactions, as the predictors. Condition and frequency were modelled as within-subjects factors, while group as a between-subjects factor. Thus, three random effects were specified, all of which allowed for variability in the intercepts but not slopes: (1) numerical participant ID, (2) the interaction between condition and ID, (3) the interaction between frequency and ID.

The group difference of ERSPs was tested for statistical significance using permutation tests (based on EEGLAB’s condstat function; Delorme and Makeig, 2004). This approach estimates both the difference between groups of their log mean spectral power, and 95% confidence intervals (CIs) of the joint distribution thereof, such that probability of the magnitude of difference can be estimated from the joint distribution. Significance was computed at two levels, *α* = 0.05 (200 permutations) and *α* = 0.0005 (2000 permutations), to illustrate a robust test statistic.

A 2 × 2 mixed ANOVA was calculated to examine whether there was a group (ADHD vs control) × TOVA condition (frequent vs infrequent) interaction in parietal prestimulus α power on correct trials. Mauchly’s test indicated that the assumption of sphericity had been violated for this data (*p* < 0.0005), so degrees of freedom were corrected using Greenhouse–Geisser estimates of sphericity to counteract the inflation of Type I errors.

#### RQ4

To estimate the effects of time for data split into “early” and “late” subsets, we analyzed the differences in log-PSD between frequency (4, 8, 10, 16 Hz), test half (H1, H2), time within test half (TWT: early, late), and group (ADHD, control). We used Bayesian LMM (Chung et al., 2013) with log-PSD as the DV, frequency, test half, TWT, and group (and their full factorial interactions, except for group), as the predictors. Frequency, test half, and TWT were modelled as within-subjects factors, while group as a between-subjects factor. Thus, four random effects were specified, all of which allowed for variability in the intercepts but not slopes: (1) numerical participant ID, (2) the interaction between TWT and ID, (3) the interaction between test half and ID, (4) the interaction between frequency and ID.

## Results

We first describe results from our behavioral analyses, then EEG results. In addition to what is reported in this section, we provide open access to the background details of our analyses (where ethically permissible, includes demographics and statistical tables, but not raw data) at https://doi.org/10.6084/m9.figshare.13614446.v1.

### Behavioral performance

Normed TOVA performance strongly distinguished diagnostic groups (note, results below all refer to the standard scores obtained by comparison to a normative population, where 100 is the reference value, i.e., lower values indicate more error, more RTV, etc.). Healthy controls were significantly more likely to have TOVA performance within normal limits compared with ADHD adults, as revealed by Pearson χ^2^ (exact two-sided) test of independence (χ^2^(1) = 7.66, *p* < 0.007).

**Figure 2.**
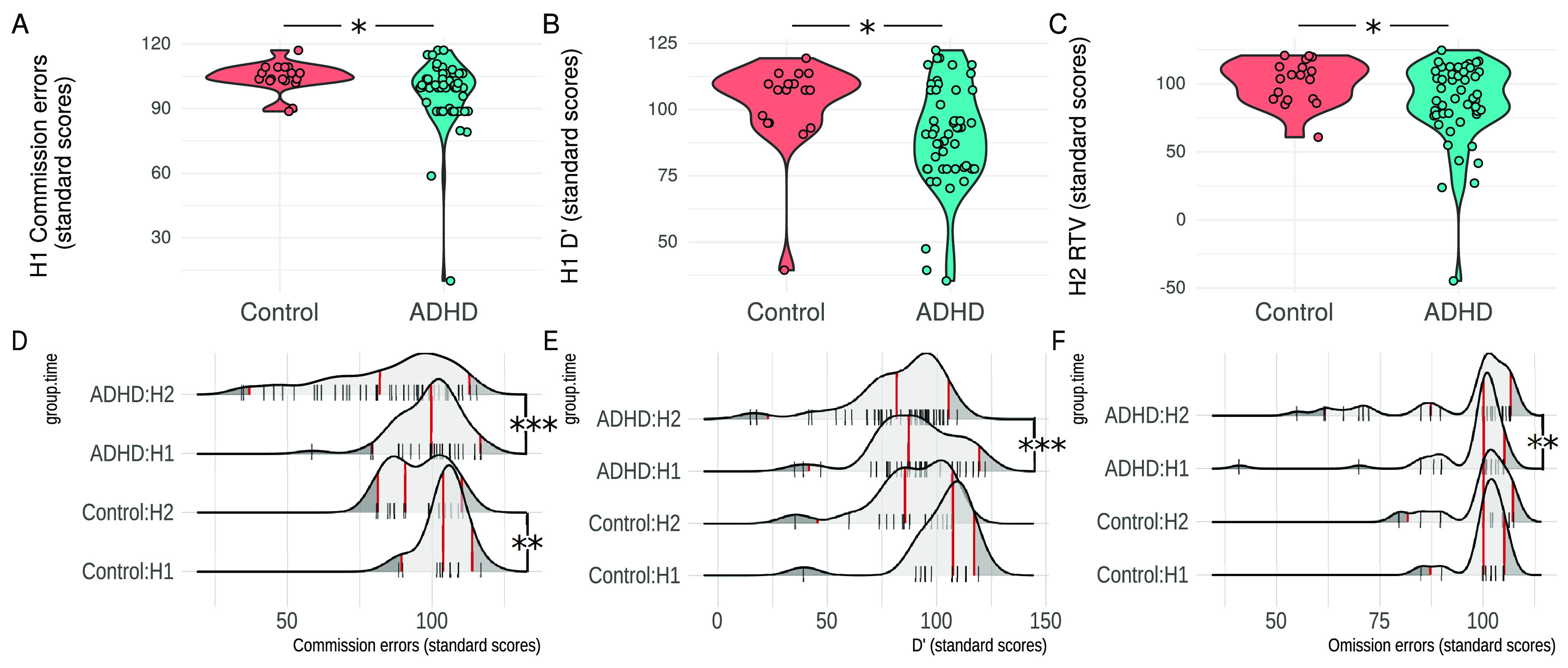
Behavioral results on standard scores (i.e., <100 implies worse performance than normative database). ***A***, ***B***, Commission errors (lower values = more errors compared with norm) and d*^′^*, from TOVA H1. ***C***, RTV from TOVA H2 (points in panels ***A–C*** shown with horizontal jitter for visibility). ***D–F***, Rug-and-density plots show commission errors, d*^′^*, omission errors, across both groups and TOVA halves. Red vertical lines show the data mean, 2.5%, and 97.5% quantiles; *p < 0.05, **p < 0.01, ***p < 0.001.

In group-wise behavioral analysis (MANOVA), there was a significant TOVA condition × group interaction (*F*_(5,61)_ = 2.63, *p* < 0.032, η^2^ = 0.177). That is, the impact that TOVA condition (H1 and H2) had on performance (as measured by the linear combination of mean RT, RTV, commission errors, omission errors and d*^′^*) depended on whether participants belonged to the ADHD group or controls. Thus, we followed up by examining the simple main effects of group and TOVA condition. Behavioral results are illustrated in Figure 2.

First, we evaluated the difference between ADHD individuals and controls at the two TOVA conditions (H1 and H2) separately, by running two one-way MANOVAs for H1 and H2. The simple main effect of group was not significant in either of these models (*F*s_(5,62)_ < 1.5, *p*s > 0.2). We then ran univariate one-way ANOVAs separately for all DVs instead of using a multivariate approach, again separately for H1 and H2. Within H1, there was a significant between groups difference (Bonferroni-adjusted for multiple comparisons) in commission errors (*F*_(1,67)_ = 4.12, *p* < 0.046, η^2^ = 0.059) and in d*^′^* (*F*_(1,68)_ = 5.30, *p* < 0.024, η^2^ = 0.074): the ADHD group tended to produce more commission errors and had a worse d*^′^* (the ability to discriminate between targets and nontargets) than the control group. Within H2, the ADHD group had significantly greater RTV (*F*_(1,68)_ = 4.52, *p* < 0.037, η^2^ = 0.064).

Next, we evaluated the difference between H1 and H2 (the difference in performance between the two consecutive conditions) separately for the ADHD and control groups, by running two repeated measures MANOVAs. There was a significant simple main effect of TOVA condition in both the ADHD (*F*_(5,44)_ = 8.96, *p* < 0.0005, η^2^ = 0.505) and control group (*F*_(5,13)_ = 7.63, *p* < 0.002, η^2^ = 0.746): both groups performed worse toward the H2 of TOVA. As before, we ran univariate repeated measures ANOVAs for each DV separately. Within the ADHD group, we found a significant effect of TOVA condition on commission errors (*F*_(1,48)_ = 26.08, *p* < 0.0005, η^2^ = 0.352), omission errors (*F*_(1,48)_ = 11.15, *p* < 0.002, η^2^ = 0.189) and d*^′^* (*F*_(1,48)_ = 20.16, *p* < 0.0005, η^2^ = 0.296): within the ADHD group performance deteriorated during the second TOVA condition across all measures. Within the control group, however, there was a significant effect of TOVA condition only on commission errors (*F*_(1,17)_ = 11.58, *p* < 0.003, η^2^ = 0.405), and d*^′^* (*F*_(1,17)_ = 8.67, *p* < 0.009, η^2^ = 0.338).

In the analysis on differences in total number of button presses, the main effect of treatment group (ADHD vs control), and the interaction between treatment group and test quarter were not statistically significant (Bs < 0.26, *t*s* *<* *1.5). Thus, there was no difference in total number of button presses between the treatment groups (on average, there were between 36.1 and 36.5 button presses for the ADHD and control groups during the first two TOVA quarters, and between 129 and 130 button presses for the groups during the last two quarters).

### EEG results

In this section, we focus on between-group analysis of frontal and parietal EEG during nonerror TOVA trials, for the following: response-locked ERPs, alignment of responses to stimulus phase, and spectral data including baseline versus poststimulus power, TFRs and ERSPs.

### RQ1: response amplitude

**Figure 3.**
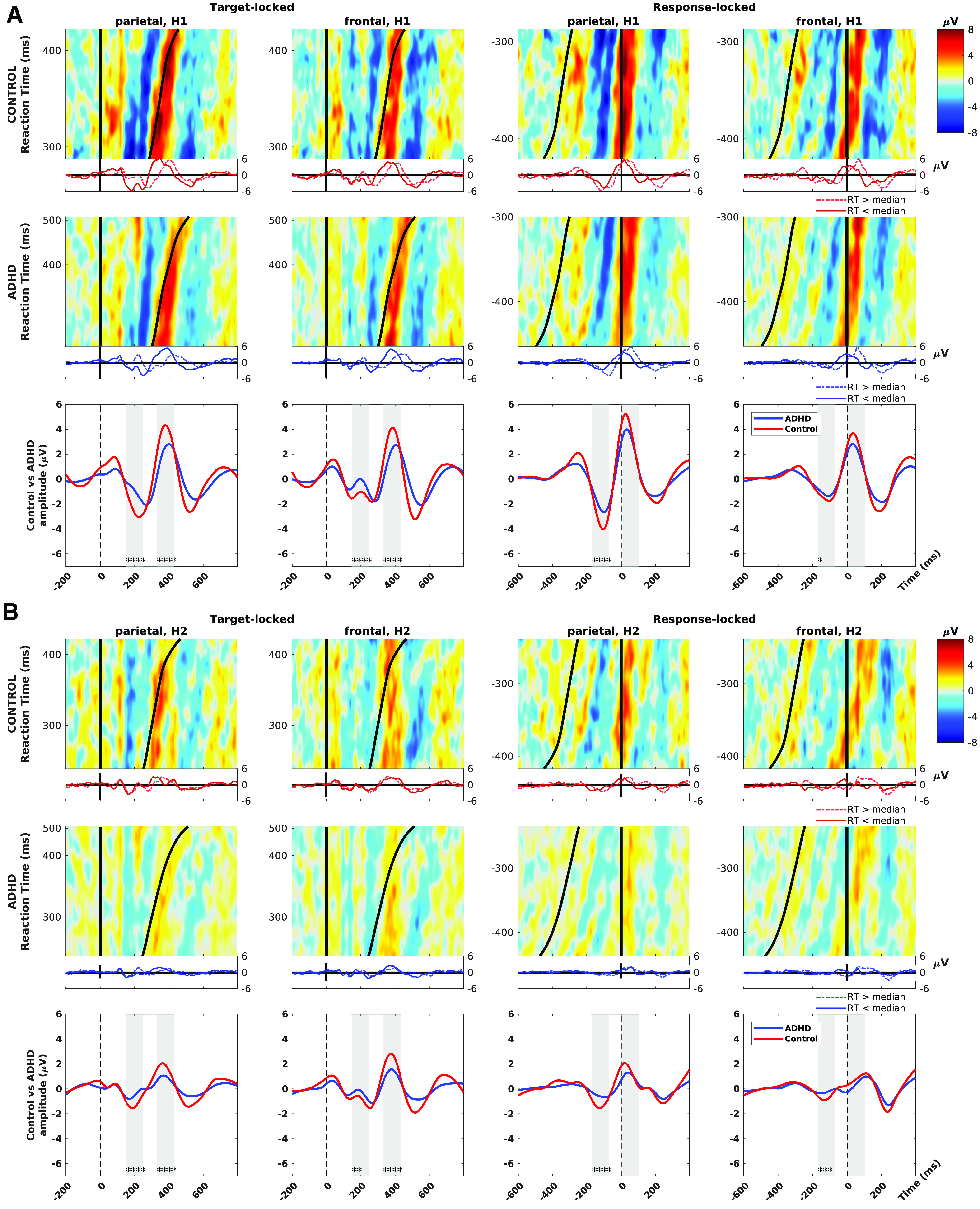
ERP image plots illustrate group-wise differences in target-locked and response-locked amplitudes. ***A***, ERP images at frontal and parietal ROIs during correct response trials during TOVA H1. ***B***, ERP images during TOVA H2. ERP images are matrices of activation data where each row is a single trial with color-coded amplitude from −8 to 8 μV (cool and warm colors, respectively). On the left are target-locked trials from −200 to +800 ms of stimulus onset and time of response shown by the black sigmoidal curve. On the right are response locked trials from −600 to +400 ms of RT, and stimulus onset shown by the black sigmoidal curve. Cumulative ERP waves are shown below each ERP image, split by the median RT. First and second row (both panels), control, and ADHD groups: these ERP images show that stimulus-locked early waves (i.e., amplitudes at a fixed lag from 0), and the response-locked waves are both much stronger for control than ADHD group. Control group’s P3 also clearly begins before the response, in contrast to ADHD group. Third row (both panels), ERP waves (solid lines) for each ROI. Vertical gray areas are test windows (aligned to N2, P3 in target-locked trials), group differences are highly significant. *p < 0.05, **p < 0.01, ***p < 0.001, ****p < 0.0001.

Figure 3 shows groupwise ERPs sorted by RT, for TOVA H1 (Fig. 3*A*) and H2 (Fig. 3*B*). Left two columns show target-locked data and right two columns show response-locked data plotted at both ROIs. In each panel, row 1 shows control group and row 2 shows ADHD, along with the grand average ERP waves for both in row 3.

As expected (Baayen and Milin, 2010), the ADHD group has more extreme RTs (>500 ms). The control group has stronger EEG responses: their maximum amplitude is ∼9 μV, while for ADHD, it is ∼6 μV; and controls have visibly stronger grand-average ERPs at both N2 and P3 (as well as earlier latency of the whole ERP in target-locked H1). Indeed, the control group P3 onset precedes the RT by a visible margin; in contrast, P3 in the ADHD group follows the RT.

Statistical testing of mean amplitude in windows (vertical gray areas in each panel’s third row), shows groups are significantly different in all target-locked conditions (D ≃ 0.1, *p *<* *0.01), and in pre-RT windows in all response-locked conditions.

However, the (second) response-locked window covering 0−100 ms post-RT is never significantly different, contrasting with the strong effect in the second target-locked window. The median-split ERPs below each ERP image explain this: in target-locked data, the ERP of the RT <median trials (solid lines) is comparable between-groups, but for the ERP of RT >median trials (dashed lines), ADHD lags control and is of smaller peak amplitude. In response-locked data, the median-split ERPs are more similar between-groups.

Finally, the full set of ERP images clearly illustrate a notable group difference: control participants experience much stronger phase-resetting: from early waves (P1, N2) before 200 ms, to the P3 preceding RT.

**Figure 4.**
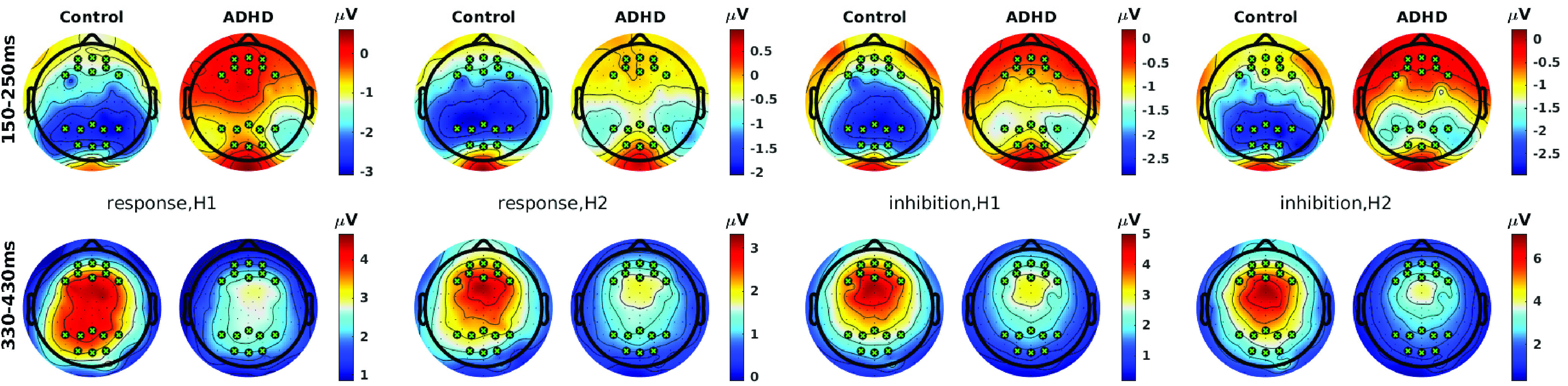
Whole-head scalp maps of broad-band amplitudes averaged within the time windows used for tests in Figure 3, for each condition: response versus inhibition × H1 versus H2. Color scales are matched to the range of the data shared between groups within each condition and time window. ROIs are shown as black and green circles.

Topographic scalp maps in Figure 4, computed within the same time windows used for tests in Figure 3, illustrate the topographic shifts in broad-band amplitude across time and between conditions/groups (as well as the exact layout of the ROIs). ADHD group activation is right-lateralized at the parietal N2 ERP.

### RQ2: phase synchrony

**Figure 5.**
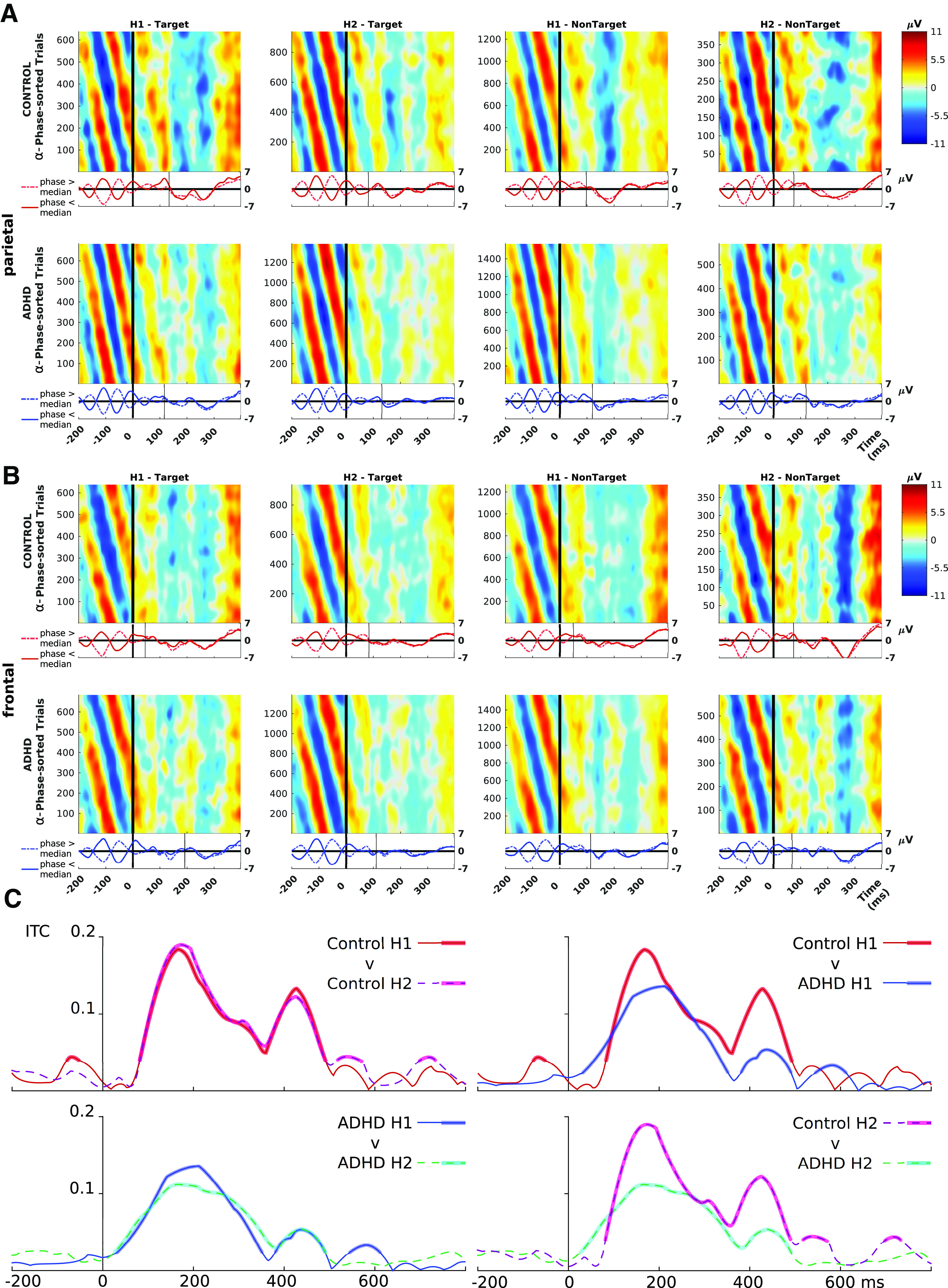
α phase-locked ERP images in both ROIs, and parietal ITC. ***A***, Phase-locked ERP images in the parietal ROI. Stacked correct-trial amplitudes are sorted by the prestimulus α (8–12 Hz) phase at −80 ms. Panels show the first and second halves of TOVA (H1 and H2), for targets (response) and nontargets (inhibition), and both groups. Amplitude from −11 to 11 μV is color-coded from blue to red, respectively. The control group shows higher amplitudes in stimulus-locked waves (starting at 100 ms), i.e., phase-resetting reaction is enhanced compared with ADHD group. ***B***, Phase-locked ERPs in all conditions in the frontal ROI. All ERP images have adjacent ERPs split at median phase, with the post-stimulus phase resetting point of alignment marked by narrow black vertical lines. ***C***, ITC calculated for both groups and both halves of TOVA, all four ITC curves show a peak around 200 ms and smaller harmonic peaks, especially at 400. Wider lines show when ITC was significantly above chance level (the level of ITC which is significant depends on the sample, so no horizontal indicator is drawn). Top left, Control group condition H1 versus H2, almost no difference between conditions is seen. Bottom left, ADHD group condition H1 versus H2, a small reduction from H1 to H2 is seen. Top right, Control H1 versus ADHD H1, substantial peak differences are seen at 200 (∼40%) and 400 (∼120%) ms. Bottom right, Control H2 versus ADHD H2, large peak differences are seen at 200 (∼90%) and 400 (∼112%) ms. Control versus ADHD comparisons also show that ADHD ITC is more dispersed, i.e., having weaker phase-locking to targets.

The picture on phase-resetting is clarified by the α phase-sorted ERP image plots in Figure 5, plotted at the parietal ROI (Fig. 5*A*) and the frontal ROI (Fig. 5*B*), for targets H1, H2 in columns 1–2, and nontargets H1, H2 in columns 3–4.

The overall control group phase reset response is clearly stronger compared with ADHD. Stimulus-locked waves beginning after 100 ms are higher amplitude (see, e.g., N2 for nontargets, or parietal P3 for targets). By splitting the adjacent ERPs at median phase, we see the prestimulus waves in opposite phase, processing to alignment at some point after stimulus onset. The groups differ in when alignment is complete (marked by narrow black vertical lines): in most conditions, controls see alignment of median phase-split ERPs before stimulus offset at 100 ms, while for ADHDs alignment happens one whole cycle later, after 100 ms.

Figure 5*C* shows the parietal 10 Hz α ITC calculated per-group and per-TOVA half. Both groups have significant 10 Hz ITC peaking at around 200 ms and its harmonics, but it is markedly stronger for control group, by around 50%.

### RQ3: α and θ dynamics

For RQ3 we first examined the log-transformed power spectral density during prestimulus and poststimulus periods of 1 s. There is a clear baseline peak, 10–11Hz parietally but close to 8 Hz frontally. In the poststimulus period, the power shifts to lower frequencies, prominent at 4 Hz. The pattern is quite similar between groups, although more pronounced for the controls.

The Bayesian LMM analysis found that the main effects of condition (*F* = 12.2, *p* < 0.001) and frequency (*F* = 173, *p* < 0.001) were statistically significant; the interaction between Condition and frequency was also highly significant (*F* = 114, *p* < 0.001), showing that log-PSD was higher during the poststimulus phase only at the 4-Hz frequency, and that at 8 Hz the order was flipped with higher log-PSD observed during the Baseline period. These findings were robust to controlling for ROIs (parietal vs frontal).

Next, we estimated the TFRs for total (ERSPs), induced, and evoked power, Figure 7, plotted across both ROIs. The data plotted here aggregates all conditions for each group, i.e., H1+H2, responses+inhibits. This was done to illustrate the gross activation patterns in each power type and ROI, and demonstrate the presence of nonphase locked power (albeit much weaker) alongside the evoked power.

The evoked power plots (third row of each panel) illustrate the phase-locking difference between ROIs, as parietal ERS (0−200 ms) is much more synchronized across frequencies than frontal ERS. The groups do not differ substantially in this (see “mean power” line plot, bottom row: evoked power is near 0 dB from 0 to 250 ms), nor does evoked power show any systematic group differences.

While the ERSP and induced power plots are very similar, the mean power line plots show how the ERSPs contain some part of the evoked power. The ERSPs, especially parietally, show the temporal extent of baseline 8-Hz spectral power shifting to 4 Hz poststimulus. Thus, next we examine ERSPs for each condition.

Figure 8 shows parietal (Fig. 8*A*) and frontal (Fig. 8*B*) baseline-corrected ERSPs for correct response and inhibition trials in H1 and H2. Conditions are ordered column-wise and groups are compared across rows (within panels), control group in row 1 and ADHD in row 2. Row 3 (in each panel) shows the between-groups differences, masked by permutation-based significance testing where gray is not significant (n.s.), lighter-toned blobs are significantly different at *p* < 0.05, and full-color blobs are different at *p* < 0.0005 (all tests uncorrected). Color in the third panel represents control ERSP minus ADHD ERSP, thus blue tones indicate ERS for ADHD.

Main patterns of difference include: ADHD group has higher prestimulus α ERS (appearing blue in the difference plots) centered on 10 Hz frontally and 12 Hz parietally, all conditions; control group has higher 8-Hz θ ERS parietally in inhibition trials; and control group has poststimulus bursts of 4-Hz θ ERS across all conditions, varying slightly in timing.

For inhibition trials, the between-groups differences in poststimulus θ is ∼1–2 dB in magnitude. Parietally the difference begins ∼100 ms after stimulus onset, across the θ band from 4 to 8 Hz, so is clearly driven by evoked power. However, this broadband power difference quickly fades while the 4-Hz difference continues at least for 400 ms. The differences in frontal θ arise later at ∼300 ms and are not broadband, suggesting frontal evoked power influence is similar between groups.

The group-wise ERSPs (rows 1 and 2 in each panel) indicate the pattern generating the differences: in all conditions the control group shows a larger initial response at all θ frequencies, which is then sustained at 4 Hz for inhibition trials. Parietal inhibition trials demonstrate the effect most clearly.

To clarify the role of parietal prestimulus α, seen in the ERSPs, our analysis for ADHD versus control showed no significant main effect of group. However, when we split the ADHD group into diagnostic subgroups: ADHD-HI/C and ADHD-I, and applied a 2 × 3 ANOVA, we found a significant but small main effect of group on parietal α power (*F*_(10,122)_ = 2.35, *p* < 0.014, η^2^ = 0.162). The between-groups difference lay between control and ADHD-I groups at 10–11 Hz (Bonferroni adjusted *p* < 0.05), wherein the ADHD-I group’s oscillatory power was twice as high (M = 9.3, SD = 6.2) as for the control group (M = 4.5, SD = 3.6). The effect was controlled for outliers defined as studentized residuals exceeding ±3. Thus, trait inattention is related to excess prestimulus α.

### RQ4: effect of time

The analysis of time within test half found no significant effects of early versus late data. Significant main effects of test half (*F* = 6.7, *p* < 0.001) and frequency (*F* = 175, *p* < 0.001), were trivially expected given the nature of the data. Of greater interest, the interaction between test half and frequency was also significant (*F* = 36, *p* < 0.001), showing that the difference in log-PSD between test halves (H2 higher than H1) was prominent only at the 10-Hz frequency.

Interesting effects of TOVA half are visible in the results plotted for earlier RQs. As noted, these should be taken with caution because of the task confound, so we first note those results that are most likely because of TOVA task.

**Figure 6.**
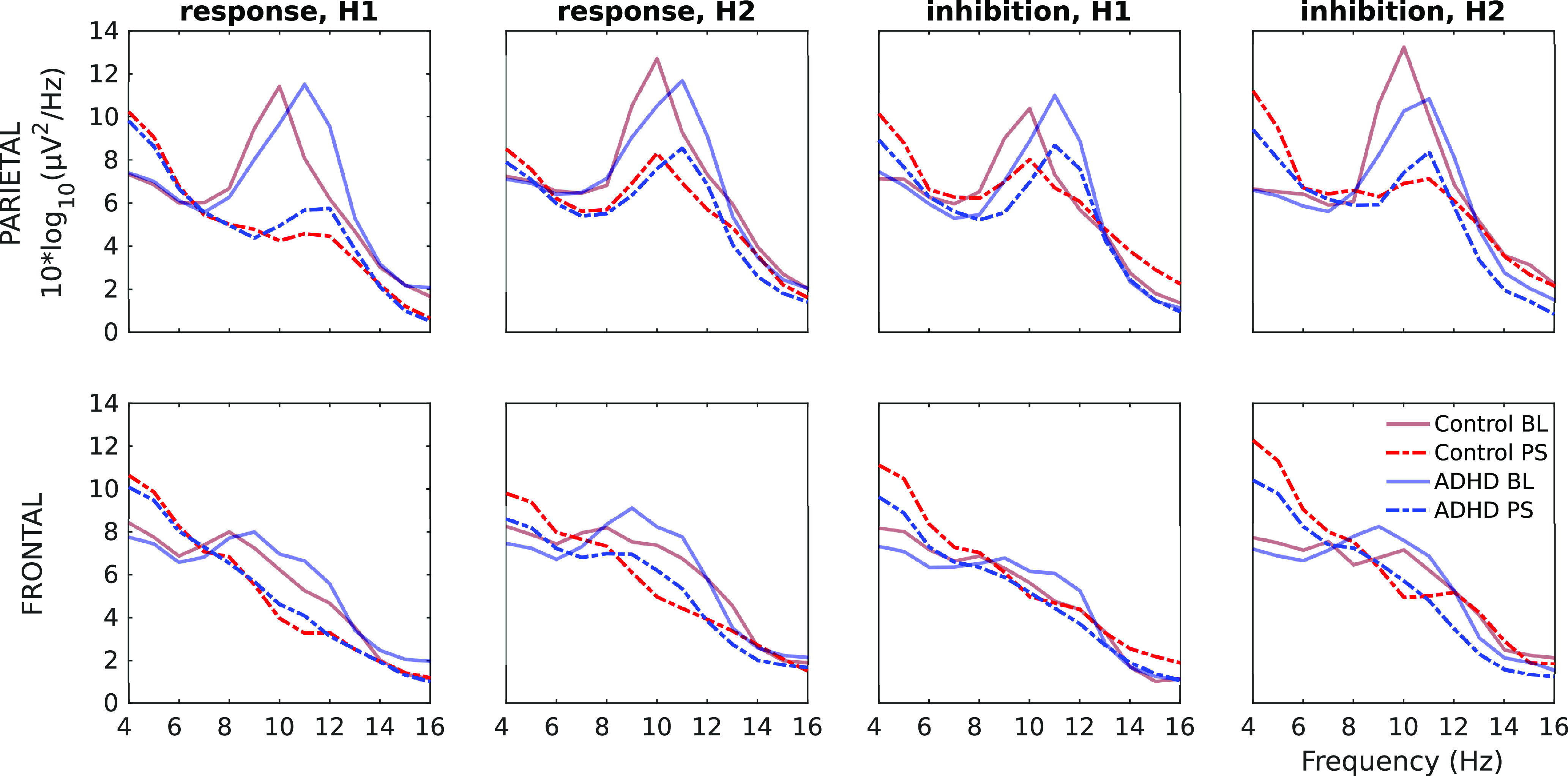
Log spectral power density calculated during periods of baseline (BL; solid lines) versus poststimulus (PS; dashed lines), for control (red) versus ADHD (blue). Panels show each condition by ROI combination. Frontally and parietally there is a higher-frequency to lower-frequency shift from baseline to poststimulus: parietal baseline peaks are α, whereas frontal peaks are at 8 Hz; in both ROIs the poststimulus shift is toward 4 Hz.

In the ERP data (Fig. 3), overall amplitude drops (H1 to H2) across ROIs and groups in both target-locked and response-locked data. In the phase-sorted ERP images (Fig. 5), for responses to targets the stimulus-locked (i.e., >100 ms) amplitudes decline from H1 to H2 (both groups, ROIs), while for nontargets the amplitudes (especially of P3) increase. In the spectral data (Fig. 6), the shift of power from BL to PS reduces in strength (from H1 to H2) for responses, but increases for inhibition. And in the ERSP (Fig. 8), the H1-to-H2 difference in ERSP strength depends on condition: responses weaken while inhibits strengthen (in both ROIs). All these results can be explained because there are fewer responses in H1, and fewer inhibits in H2, thus giving them an “oddball” quality which may enhance neural activation, for example, the ERP data are from response trials so the activation might be stronger in H1 when responses are infrequent.

However, there are a few results which are not explained by this frequency effect. For the ERP data (Fig. 3), comparing H2 to H1, the RT median-split ERPs become less distinct from each other; the target-locked grand-average ERPs of each group align more in latency; and the response-locked activations are less aligned to the response but follow the stimulus onset more uniformly in time (visible as a strong “flattening” of the frontal H2 ERP). All this suggests an effect of habituation and automatization of responding. In the ITC data (Fig. 5), the ADHD group peak ITC dropped from H1 to H2, and the temporal extent of significant-ITC spread out, to begin earlier and end later. In contrast, for controls both H1 and H2 had almost equal profiles of significant ITC, suggesting they maintained greater consistency across halves. Finally, in the ERSP (Fig. 8), H1 to H2 change in strength of statistical difference depends on ROI: parietal effects seem to weaken, while frontal effects strengthen.

## Discussion

The present study explored cortical oscillations and event-related activations during sustained attention in adults with ADHD and in a healthy control group, measuring EEG while they performed the TOVA task.

### TOVA performance results

In terms of behavioral performance within halves, the ADHD group was affected by task demands in line with expected deficits. During H1 (infrequent targets testing inattention), the ADHD group had more commission errors and a lower d*^′^* than the control group; suggesting they did not maintain task focus. During H2 (frequent targets testing inhibition) they had more variable RTs than controls, suggesting deficient prediction of target onset in line with Russell et al. (2006).

Both groups performed worse as the test progressed, with decreased d*^′^* (possibly because of reduced vigilance) and increased commission errors (possibly because of fatigue). However, for the ADHD group the amount of omission errors also increased during H2, possibly because of mind wandering as suggested by the cognitive-energetic model of ADHD (Sergeant, 2000, 2005; Killeen, 2013).

### RQs

Regarding RQ1, controls had early ERP waves with significantly greater amplitude than the ADHD group (Fig. 3); their P3 waves also coincided with responses, compared with ADHD P3s which typically followed responses. For RQ2, the control group showed stronger phase-resetting in response to target stimuli, and control ITC profiles were both more focal and 50% stronger at peak (Fig. 5). Taken together, this evidence shows that ADHD participants were more weakly tuned to the periodic temporal profile of the TOVA trials, and thus had diminished capacity to predict the onset of the next stimulus, as expected (Russell et al., 2006).

Amplitude scalp maps in Figure 4 also reflected parietal asymmetry at the N2 ERP, in line with prior findings on N2 (Gruendler et al., 2011). Indeed right-biased cortical asymmetry in ADHD has been a common finding, for example in θ band among adults (Hale et al., 2014; Jang et al., 2020). Hale et al. (2014) suggested that “atypical rightward asymmetry should be broadly reflective of any form of nonoptimized task-directed brain functioning,” which we elaborate on below.

RQ3 ERSP results show two main patterns. First, the control group had significantly higher prestimulus ERS parietally at 8 Hz and poststimulus 4 Hz ERS during inhibition trials in both ROIs. Landau et al. (2015) examined parietal θ in the context of visual discrimination-task performance, establishing a precise relationship between two-target spatial attention and γ-band activity phase-modulated by a parietal 4-Hz source. More recently, Spyropoulos et al. (2018) showed that (in macaque V4) parietal θ plays a role in modulating γ-frequency coding of visual input, and in thus mediating visual attention. Evidence shows that sustained attention is a rhythmic sampling process occurring at a base rate of 8 Hz when monitoring a single stimulus; this sampling rhythm decreases to 4 Hz when there are two targets to monitor, and keeps decreasing with increasing number of targets (Holcombe and Chen, 2013). Observation of these effects in human electrophysiology indicates that they could be found also in scalp EEG, if generated strongly. TOVA sequentially presents two spatially-distinct (but otherwise identical) stimuli in a distraction-free context, thus providing ideal conditions to generate strong ∼4-Hz parietal θ ERS, exactly as observed. The shift in control group parietal trials, from 8-Hz prestimulus to evoked band-wide and then 4-Hz ERS (which is also frontal), also supports this interpretation. Similar sensor-level activation patterns were observed for divided attention by McCusker et al. (2020).

In the second ERSP result, the ADHD group had significantly higher α ERS across the prestimulus period, in both ROIs in all conditions except H2 inhibition. This observation is in line with several prior studies that found deficient α suppression in ADHD during visual attention tasks (Lenartowicz et al., 2018).

**Figure 7.**
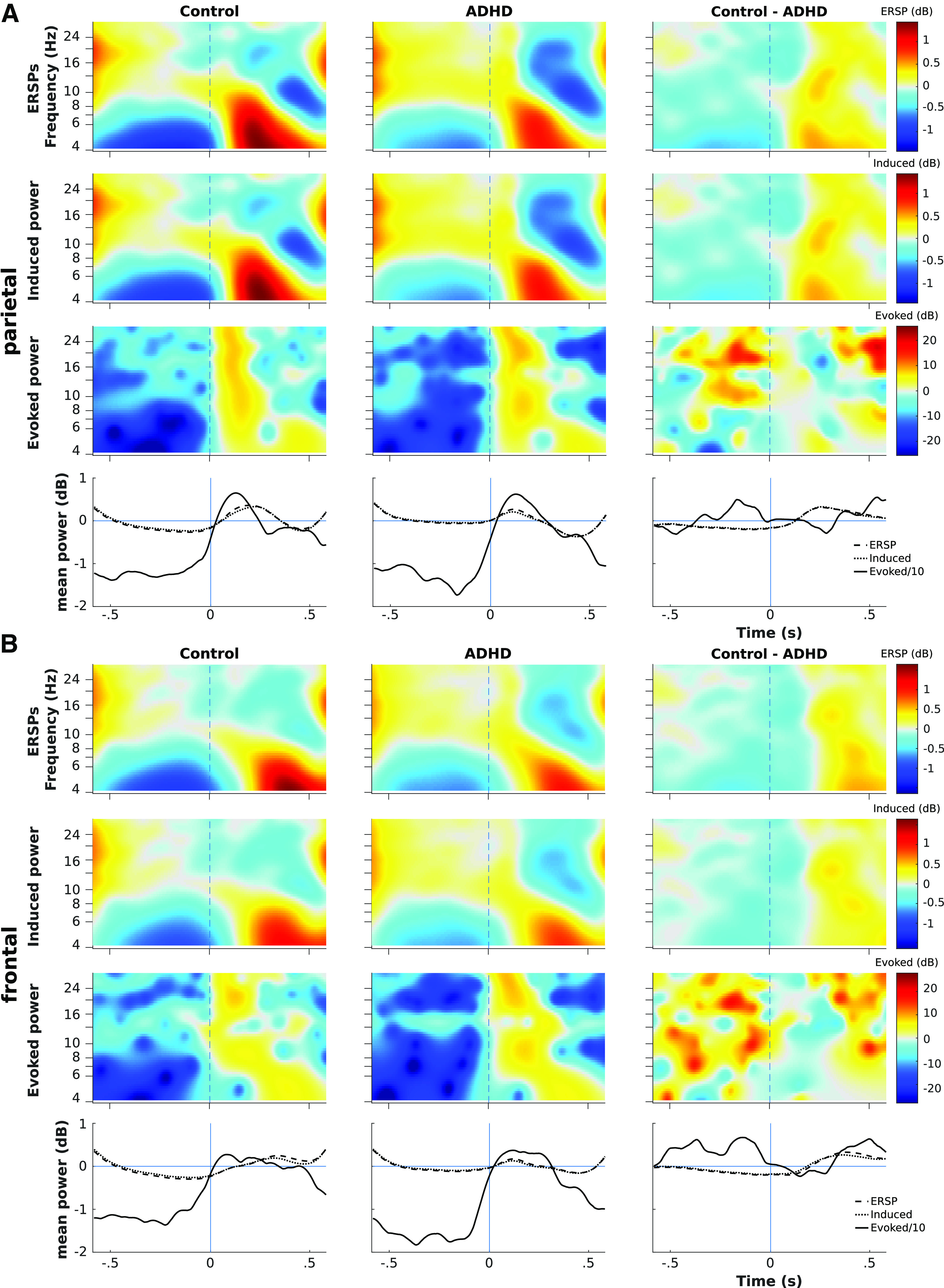
Contrasting groups for ERSPs, induced power, and evoked power. ***A***, TFRs plotted within parietal ROI. ***B***, TFRs plotted in frontal ROI. Each TFR is averaged across all electrodes in the ROI; TFRs are locked to stimulus onset at time 0 (dashed vertical lines); frequencies are plotted on a log scale. Within each panel, first row shows ERSP, second shows evoked power, third shows induced power, and fourth row shows the mean across frequencies of the upper three (with evoked power divided by 10 to illustrate). Left and middle columns show ERS/ERD for control and ADHD groups, respectively; the right column shows the subtracted difference between groups for the ROI-averaged TFR (or its mean in row four).

It is of note that, while there were large differences in evoked power in between groups (Fig. 7, row 3), the differences had no systematic pattern and thus provide no grounds for interpretation.

Loo et al. (2009) found increased cortical arousal in the ADHD group during Conners’ CPT (Conners et al., 2000), indicated by attenuated frontal and parietal α power. This effect increased toward the end of the task. However, they did not find any differences between groups in θ power. Our ERSP and prestimulus α power analyses both contrast with Loo et al. (2009). θ Was systematically different between groups, and ADHD group did not exhibit relatively lower parietal α power. Indeed, we found that the ADHD-I subgroup’s parietal 10- to 11-Hz α power was twice that of the control group. This implies that these participants exerted less cognitive control and had diminished α suppression during TOVA. It is of note that differences were found only at 10–11 Hz, precisely in time with the speed of presentation of TOVA stimuli. Given that this occurred parietally, it may relate to the observation of weaker (attention-sampling) parietal θ.

Finally, most RQ4 results were attributed to the task confound; the remaining results distinguished the controls as maintaining task focus, for which they anyway had an advantage (in terms of phase synchrony with stimuli and strength of response) even in H1. Thus, the deterioration of performance over TOVA halves appears a normal consequence of fatigue, brought on by paying sustained attention over an extended period of time.

Taken together, the results of RQ1–RQ4 indicate that in TOVA, ADHD participants were mainly affected by trial-wise deficits, and not a deficiency of long-term neural energetics (Killeen, 2013), this might reflect that the task duration is well within the capabilities and self-efficacy expectations of even adults with ADHD but that performance still suffers because of some deficiency of trial-wise processing, which we address next.

### Interpretation of results

Synthesizing all observed results, we propose an interpretation based on deficient rhythmic attention-sampling in a cortical area responsible for relational processing, the posterior parietal cortex (PCC). This may be inferred from the nature of the TOVA task itself, as follows.

The TOVA task is temporally regular, such that every trial has predictable onset. This can be exploited to study ADHD by comparing behavioral and neural responses to neurotypical responses which have been facilitated by predictability. Our data suggest the ADHD behavioral deficit arises from a dual difference to controls in θ and α. θ Is reduced parietally at 8-Hz prestimulus and band-wide stimulus-locked, and fronto-parietally at 4 Hz from 200 ms on; α, by contrast, is increased fronto-parietally in the prestimulus period, especially in H1.

We suggest these results are possibly because of weaker endogenous attention sampling, specifically, in line with the theory that attention continuously samples attended locations at an 8 Hz base rate (Landau et al., 2015). This theory also states that monitoring multiple locations reduces to attention-sampling rate to some fraction of 8 Hz, e.g., 4 Hz for two locations (Holcombe and Chen, 2013). Our data suggest that the weaker θ and stronger α parietal prestimulus activations actually cause the observed weaker phase resetting in ADHD. The activations may reflect excessive modulation of endogenous activity by strong entrainment to stimulus (α), combined with deficient modulation by neural entrainment to task (θ), which in this case is monitoring stimulus spatial location (not their predicted occurrence onset which is regular and task-irrelevant). This consequently leads to weaker early ERPs (see Fig. 3) and smaller peak ITC (see Fig. 5). Thus, ADHD may have poorer TOVA performance because of weaker modulation of attentional sampling (especially for stimuli requiring inhibition), reflected (for example) as significantly higher commission error rate in H1 (frequent nontargets). The effect could be likened to watching the hedgerows zip by while driving, instead of monitoring for road signs.

Further, TOVA is a relational classification task: Go/NoGo (respond/inhibit) to targets/nontargets, which share the same stimulus properties (smaller black rectangles within larger white squares), at two different spatial locations (above-below fixation). This relationship between target types permits a unidimensional encoding, and θ has indeed been proposed to facilitate relational encoding (Lisman and Jensen, 2013). Based on this property of the task, we can hypothesize a cognitive mechanism that could generate our above results, as follows.

Summerfield et al. (2019) recently described a theory for the role of PPC (i.e., part of the dorsal visual stream, the so-called “what” stream) in processing relational structure in the environment. In brief, Summerfield et al. (2019) suggest that primate PPC supports learning and processing of relational structure, for example in visual scenes. They build on work establishing that PPC, in particular lateral intraparietal area (LIP; Brodman’s areas 39/40), provides spatially-selective coding for regions of ego-centric space that subserve functions including decision-making and top-down spatial attention (Freedman and Ibos, 2018). PPC neurons also code for abstract categories (Freedman and Assad, 2016), in a scalar manner wherein unidimensional relations are coded asynchronously, if A is coded by many neurons, then B may be coded by relatively fewer. Since TOVA stimuli consist exactly of a unidimensional relational structure, every trial requires recruitment of the PPC to judge the presented relationship and trigger appropriate action.

Finally, the observed difference in 4-Hz θ ERSP frontally suggests an involvement of executive function, which gets stronger from H1 to H2. In Figure 5*B*, the frontal ADHD group ERPs, split by median phase, show how phase alignment after stimulus onset happens earlier in H2 than H1 for both conditions. It seems that as ADHD participants become habituated to the TOVA trials, their frontal 4-Hz task monitoring rhythm weakens, and as a possible consequence, Figure 3*B* shows how their P3 becomes more locked to stimulus onset than to RT.

Our ultimate hypothesis is that our ADHD group show deficient θ-rhythmic attention sampling, linked to the specific function of sampling relationships in the world. Interesting converging evidence was presented by (Jang et al., 2020) for adults with ADHD traits, i.e., ADHD was not clinically diagnosed but identified by self-report (Conners’ Adult ADHD Rating Scales). Subjects performed a spatial two-back task (bringing a working memory demand), with similar trial timing and amount as TOVA, but with nine spatial locations to attend. (Jang et al., 2020) also calculated ERSP (but based on Fourier transform, not wavelets), and averaged the power across frequency bands and in frontal and parietal ROIs (although with fewer channels). They observed somewhat similar results to ours, with weaker θ and stronger α in the ADHD-trait group, and found faster RTs were associated with increased parietal θ power for the controls but not ADHD-trait. They did not publish ERSP visualizations or data on temporal evolution of specific frequencies, but their results do support the interpretation of a deficit in spatial relational coding.

One question that arises is the following: why was the significant difference in between-groups ERSP to response trials much weaker? If we draw again on the Summerfield et al. (2019) theory, and the scalar coding of unidimensional relations, the interpretation would be that ERS to response trials is generally weaker because of having fewer coding neurons, and thus between-group differences get averaged out because of lower signal-to-noise ratio.

Alternative explanations are also possible. For one thing, primate data (Fitzgerald et al., 2013) supporting the above theory shows that individuals code category classes arbitrarily, and the group-wise aggregate in our data should then “even out,” i.e., higher firing for targets in some participants, and vice versa in others. If this is the case, our results would imply that (especially inhibition) trials differed in some other way than PPC coding. One option is that ADHD θ ERS was spread uniformly across frequencies and thus not strong enough at the monitoring frequency 4 Hz, because ADHD participants were not sufficiently focused on attending the correct spatial location, and this effect was stronger for inhibition than response trials because responses have action affordances, which can leverage premotor cortex coding to supplement PPC category judgment. However, the first explanation is more parsimonious.

### Limitations

Since the aim was to study the neural correlates of TOVA in particular, we were thus bound to the particular design of that test, which has some atypical features compared with other lab tasks for attention: notably the fixed task structure and periodic stimulus presentation. As such, there were a number of limitations, both in the sense of constraints and issues.

### Constraints

The foremost constraint on analysis is that time dependency is confounded by the fixed order of TOVA tasks in H1 and H2, which are not counterbalanced. Of course, such bursty temporal structure is quite natural and of interest (Karsai et al., 2012); however, future work should aim to adapt TOVA to counterbalanced presentation.

The small size of some of the TOVA norming groups, such as males aged 30–39 (*n* = 4), might have increased the number of false positives. In the present study, 19 participants (of which 15 with ADHD) belonged to this cohort. The low sample sensitivity and specificity highlight the issue of behavioral heterogeneity among people diagnosed with ADHD, and further motivates our sub-group analysis which found elevated parietal 10 Hz α among the ADHD-I group (compared with controls).

**Figure 8.**
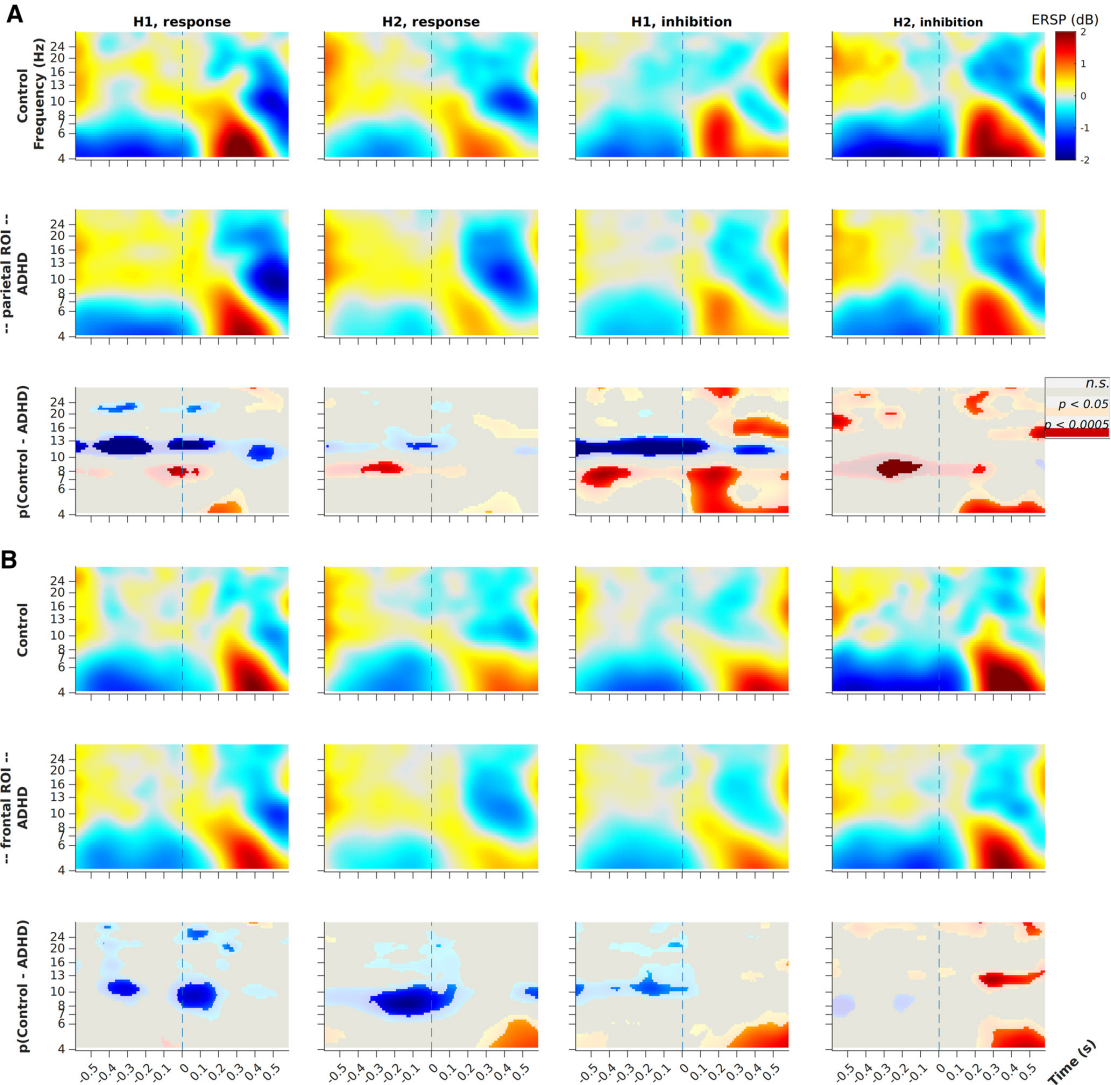
ERSP plots for condition: correct inhibition versus response trials, in H1 versus H2. ***A***, ERSPs plotted in the parietal ROI. ***B***, Frontal ROI ERSPs. The time-frequency data are averaged across all electrodes in the ROI; the scale of power perturbations goes from −2 to 2 dB; ERSPs are locked to stimulus onset at time 0 (dashed lines); frequencies are plotted on a log scale. Within each panel, each condition is plotted column-wise. Top and middle rows show ERS/ERD for control and ADHD groups, respectively. Bottom row shows the log-mean difference between groups for the ROI-averaged time-frequency data; these plots are masked by a permutation-based significance test: gray is non-significant (n.s.), lighter-toned blobs are different at *p* < 0.05, and full-color blobs are different at *p* < 0.0005 (all tests uncorrected).

In terms of constraints of the task, TOVA does not aim to induce a large number of error trials, which limited the types of analyses available. For example, we could not subtract correct response trials from commission error trials, or vice versa. As a result, the ERSP plots for correct response trials (Fig. 8) contain not only cognitive processing but also motor processing related to the response.

In using TOVA to study oscillations instead of evoked responses, we started from the assumption that the brain is predictive (Sherman et al., 2016), and thus the periodic structure of the task facilitates the involvement of endogenous rhythms. In fact, such predictions are logically required, because the TOVA task requires rapid distinction of two random (but not equiprobable) alternatives, so competing prediction “cascades” must be launched to prepare for either motor response, or inhibition. Against the idea that all activity during a task like TOVA represents evoked activity, with between-stimulus activity simply “driven” by the periodic stimulus presentations, there is empirical (Zoefel et al., 2018) evidence for predictive processing that suggests the brain is not simply receptive this way, and theoretical (David et al., 2006) evidence that the recorded activity anyway represents a mixture of effects.

### Issues

In order to recruit sufficient *N*, the present study had a sample with a wide range of ages. There are well-documented age-related changes in tonic α and θ oscillations; for example, the age-related evolution of α and θ frequency spectra is nonlinear and may lead in opposing directions for α and for θ (Klimesch, 1999). However, given that our analyses were conducted within (not between) frequency bands, and the age range of ADHD and control groups were balanced, we do not believe this issue alters our interpretation of results.

We did not control for individual differences in α peak frequency (iAPF), that is, the single frequency in the α band showing the highest power per individual. Counting iAPF as the central frequency of α, instead of 10 Hz, can be used to recalibrate all frequency bands and potentially account for clinical variation in EEG spectra (Lansbergen et al., 2011). However, the technique might lead to an additional filter-effect confound in analysis of such short time-scales as TOVA trials, so was avoided in favor of a clearer processing pipeline.

The neuropathological heterogeneity of ADHD implies that distinct neurocognitive subtypes of ADHD may exist, and yet not map to symptomatic subgroups. Because of this, one criticism of ADHD studies has been that group-wise comparisons between ADHD participants and healthy controls are likely to yield small, hard-to-replicate effects (Nigg et al., 2005). While the present study compared groups based on their diagnostic status and did find robust behavioral differences, also clustering participants as a function of both behavioral performance and cortical oscillations may find meaningful distinctions in test responses. This type of analysis-by-outcome (as opposed to analysis-by-diagnosis) might shed more light on how oscillatory dynamics are related to performance, and how this relationship is altered in ADHD as compared with healthy controls.

In conclusion, this study is among the first of its kind combining TOVA with an EEG measurement in adults (Keith et al., 2015) – and the first to focus on ADHD. We showed that the ADHD group had increased prestimulus α ERS, reduced θ ERS during correct inhibition trials, and demonstrated reduced sensitivity to stimulus timing in phase-resetting and ITC. Our interpretation of these results in terms of parietal θ coding of relational structure points to deficient attention sampling in relational categorization tasks (Summerfield et al., 2019). This result contributes to the overall understanding of the neuropathology of ADHD in adults.

Both the clinical and scientific pictures of ADHD remain complex and multidimensional (Killeen et al., 2011). Because of this, it is important to search for neurocognitive mechanisms that explain aspects of the disorder, mutual information from multiple mechanisms (e.g., behavioral and neural) could improve treatment targeting efficacy (Arns et al., 2008). The use of CPT with EEG measurement for assessing neural correlates of ADHD in adults is understudied, and combining EEG measurement with TOVA has the potential to explain why differences in behavioral performance in TOVA arise.

## Synthesis

Reviewing Editor: Satu Palva, University of Helsinki Decisions are customarily a result of the Reviewing Editor and the peer reviewers coming together and discussing their recommendations until a consensus is reached. When revisions are invited, a fact-based synthesis statement explaining their decision and outlining what is needed to prepare a revision will be listed below. The following reviewer(s) agreed to reveal their identity: NONE.

In this revision, the points raised in the previous review have largely been addressed or become moot by the removal of phase synchrony analyses. The manuscript has overall improved considerably and is easier to understand now. However, the points raised about the nature of observed activity are not answered sufficiently. The argumentation that predictive brain activity is largely oscillatory has its charm, but authors should have a good justification why there are presumably no large group differences in evoked activity in the discussion section.

Lines 89-101: This part is now largely formulated such as it were part of the Discussion. This should be changed to some short statement like “while TOVA has traditionally been used to study evoked responses, we here posit that [...] and therefore TOVA can also be used to study oscillations", and the rest should be moved to the Discussion.

Lines 126-128: were unequal numbers of participants compared?

Lines 239-240: “This ... ensured each participant contributed 108 clean epochs per condition.” How exactly does this follow from the previous sentence?

Lines 240-241: “The epochs were selected at random” - it is not quite clear here what exactly was done. How many epochs were selected out of how many available ones? Was this carried out only once? Depending on this ratio, it might be interesting to check if results are consistent across different draws.

Lines 392-410 & Figure 2: While I understand from the rebuttal that the standard commission errors are designed so that higher values represent better performance, i.e. less errors, this is still not made clear in the results or in the figure legend. In my opinion, it should be stated explicitly in both. Else many readers will be confused, as such a scoring system for errors is somewhat counter-intuitive.

Figure 4 should state the amplitudes represent broadband activity.

Lines 615-616: Please reformulate, results are not due to a theory.

## Author Response

Second round of reviews for manuscript “Reduced power in fronto-parietal theta EEG linked to impaired attention-sampling in adult ADHD"

Thank you for the further comments on our manuscript. We’re glad that you found

our revisions largely satisfactory; our responses to the further points raised are listed below. We have also revised our manuscript accordingly.

Comments from the Editor and the viewer:

[...] However, the points raised about the nature of observed activity are not answered sufficiently. The argumentation that predictive brain activity is largely oscillatory has its charm, but authors should have a good justification why there are presumably no large group differences in evoked activity in the discussion section.

Response: In fact there are very large differences in evoked activity. Figure 3 shows much larger target-locked ERP amplitudes for Controls than ADHD. Figure 7 shows very strong group differences in evoked power (third row in each panel) -- it just happens to not be systematic at all, thus offering little in the way of interpretable insight.

In our view, it is not that there are no evoked differences, but rather that the most interesting results (from the ERSP) are best explained in view of the oscillatory arguments put forth. We have a short note in the Discussion to this effect.

Lines 89-101: This part is now largely formulated such as it were part of the Discussion. This should be changed to some short statement like “while TOVA has traditionally been used to study evoked responses, we here posit that [...] and therefore TOVA can also be used to study oscillations", and the rest should be moved to the Discussion.

Response: We have edited these paragraphs largely as suggested; one entire paragraph was moved to the discussion while the other was clarified. We could not state that TOVA has been traditionally used to study evoked responses, as there does not seem to be a strong tradition of using TOVA with EEG/MEG

Lines 126-128: were unequal numbers of participants compared?

Response: Indeed, each condition had slightly different numbers of participants, simply because the amount of artefactual data varied by condition. Given that each condition retains a suitable sample size, and that

we are not interested in comparing conditions (but rather groups), this was not felt to be a problem.

Lines 239-240: “This ... ensured each participant contributed 108 clean epochs per condition.” How exactly does this follow from the previous sentence?

Response: There was a slight error in the text, which we have now fixed. The text now reads (changes in bold):

"This resulted in the participant numbers given above, and ensured each participant contributed 108 clean epochs per each test half (H1 and H2; i.e. responses H1 with 36 epochs + inhibits H1 with 72 epochs, totalling 108 epochs, and similarly for H2).

Lines 240-241: “The epochs were selected at random” - it is not quite clear here what exactly was done. How many epochs were selected out of how many available ones? Was this carried out only once? Depending on this ratio, it might be interesting to check if results are consistent across different draws.

Response: We have clarified this method, see lines 238-241. The epochs were randomly sampled without replacement from the total available per condition, on a participant-wise basis. The total epochs available per condition varied by participant. Sampling was done once, to provide a fixed representative sample of epochs for all analyses. However, the overall effect of resampling was minimal, as showed by additional analysis of spectral data which we reported numerically (but figures for which can be seen below

Lines 392-410 & Figure 2: While I understand from the rebuttal that the standard commission errors are designed so that higher values represent better performance, i.e. less errors, this is still not made clear in the results or in the figure legend. In my opinion, it should be stated explicitly in both. Else many readers will be confused, as such a scoring system for errors is somewhat counter-intuitive.

Response: We have included more detailed descriptions of this in the

Methods, Results, and figure caption.

Figure 4 should state the amplitudes represent broadband activity.

Response: This has been changed as requested

Lines 615-616: Please reformulate, results are not due to a theory.

Response: True. We now write “ ...in line with the theory that attention... “
